# Disparities in time to prostate cancer treatment initiation before and after the Affordable Care Act

**DOI:** 10.1002/cam4.6419

**Published:** 2023-08-03

**Authors:** James R. Janopaul‐Naylor, Taylor J. Corriher, Jeffrey Switchenko, Sheela Hanasoge, Ashanda Esdaille, Brandon A. Mahal, Christopher P. Filson, Sagar A. Patel

**Affiliations:** ^1^ Department of Radiation Oncology Winship Cancer Institute at Emory University Atlanta Georgia USA; ^2^ Department of Radiation Oncology Memorial Sloan Kettering Cancer New York New York USA; ^3^ Department of Biostatistics and Bioinformatics Rollins School of Public Health Atlanta Georgia USA; ^4^ Department of Urology Emory University School of Medicine Atlanta Georgia USA; ^5^ Department of Radiation Oncology University of Miami Miller School of Medicine Miami Florida USA

**Keywords:** ACA, disparities, Obamacare, prostate cancer, treatment delay

## Abstract

**Background:**

Delayed access to care may contribute to disparities in prostate cancer (PCa). The Affordable Care Act (ACA) aimed at increasing access and reducing healthcare disparities, but its impact on timely treatment initiation for PCa men is unknown.

**Methods:**

Men with intermediate‐ and high‐risk PCa diagnosed 2010–2016 and treated with curative surgery or radiotherapy were identified in the National Cancer Database. Multivariable logistic regression modeled the effect of race and insurance type on treatment delay >180 days after diagnosis. Cochran–Armitage test measured annual trends in delays, and joinpoint regression assessed if 2014, the year the ACA became fully operationalized, was significant for inflection in crude rates of major delays.

**Results:**

Of 422,506 eligible men, 18,720 (4.4%) experienced >180‐day delay in treatment initiation. Compared to White patients, Black (OR 1.79, 95% CI 1.72–1.87, *p* < 0.001) and Hispanic (OR 1.37, 95% CI 1.28–1.48, *p* < 0.001) patients had higher odds of delay. Compared to uninsured, those with Medicaid had no difference in odds of delay (OR 0.94, 95% CI 0.84–1.06, *p* = 0.31), while those with private insurance (OR 0.57, 95% CI 0.52–0.63, *p* < 0.001) or Medicare (OR 0.64, 95% CI 0.58–0.70, *p* < 0.001) had lower odds of delay. Mean time to treatment significantly increased from 2010 to 2016 across all racial/ethnic groups (trend *p* < 0.001); 2014 was associated with a significant inflection for increase in rates of major delays.

**Conclusions:**

Non‐White and Medicaid‐insured men with localized PCa are at risk of treatment delays in the United States. Treatment delays have been consistently rising, particularly after implementation of the ACA.

## Graphical abstract

The Affordable Care Act (ACA) attempted to increase access to care and reduce healthcare disparities. However, we show that major delays (>6 months from diagnosis to starting treatment) for prostate cancer were more prevalent after the ACA was implemented, with Black patients having almost twice the odds of experiencing a major delay. Furthermore, patients with Medicaid were just as likely to experience major treatment delays as uninsured patients after the ACA, while patients with private insurance or Medicare had lower odds of treatment delays.

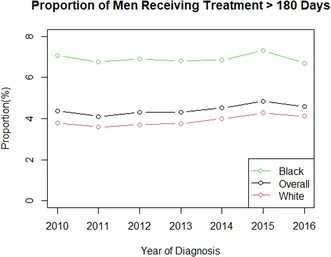

## 1 INTRODUCTION

In 2022, there will be over 260,000 new cases of prostate cancer in the United States.^1^ Black men, compared with White men, are more likely to be diagnosed with and die of prostate cancer.^2^ The root of these disparities is multifactorial, possibly due to intrinsic tumor biology, systemic racism, as well as medical mistrust.^3^, ^4^, ^5^, ^6^, ^7^, ^8^, ^9^, ^10^ Recent studies, however, suggest that racial disparities in prostate cancer outcomes in the United States may be driven predominantly by disparities in access to care and receipt of definitive therapy.^11^, ^12^, ^13^ Specifically, Black men have historically experienced significantly longer delays from cancer diagnosis to treatment initiation compared with White men.^14^

In the last decade, multiple structural changes have affected the management of prostate cancer as well as attempted to rectify disparities in access and care. In 2010, the Patient Protection and Affordable Care Act (ACA) was created to expand access to care and reduce healthcare disparities,^15^ but was not fully operationalized with personal coverage mandates, development of insurance exchanges, elimination of pre‐existing condition plans, coverage of preventative care, and more until 2014. Evidence in breast, colon, and lung cancer suggest that while screening rates and early stage detection have improved after the ACA, disparities in timely cancer treatment initiation remained.^16^, ^17^ In the metastatic setting, Medicaid expansion under the ACA was associated with decreased racial disparities for timely initiation of systemic therapy.^18^ One theory is that the ACA would disproportionately benefit non‐White patients who were uninsured, increasing their access to care through Medicaid. However, the impact of the ACA on timely access to prostate cancer care is unclear, particularly as patients with Medicaid more frequently encounter providers who refuse to accept their insurance.^19^ Additionally, timely treatment initiation can be affected by patient preferences, shifting prostate cancer epidemiology, and lagging changes in the healthcare labor force to accommodate for potential increases in patient volume. In this analysis, we evaluated the impact of the ACA on timely access to care for men with intermediate or high risk localized prostate cancer in the United States, with a focus on its impact based on race.

## 2 METHODS

### 2.1 Data source and study population

The National Cancer Data Base (NCDB) is a nationwide hospital‐based registry sponsored by the American College of Surgeons and the American Cancer Society. It captures the first course of cancer treatment from more than 1500 Commission on Cancer‐accredited facilities, gathering data on approximately 70% of new cancer diagnoses in the United States.^20^, ^21^ Data accuracy are continually validated via quality review, site surveys, and internal monitoring.^20^, ^21^, ^22^ Because the study used de‐identified data from the NCDB, the requirement for formal institutional review and the need for informed consent were waived.

We identified men ≥40 years old with a new diagnosis of clinical stage T1‐4, N0, M0 prostate adenocarcinoma between 2010 and 2016. Those pursuing definitive treatment with radical prostatectomy or radiation therapy (with or without concomitant androgen deprivation therapy [ADT]) were eligible. We excluded patients with low‐risk disease (i.e., T1‐2a and Gleason score 6 and PSA <10), patients pursuing active surveillance or palliative therapy, patients treated with chemotherapy or immunotherapy, patients with prior or synchronous diagnosis of other malignancy, and patients with missing date of surgery or start of radiation therapy or ADT (Figure S1).

The exposure of interest was non‐Hispanic White versus other races or ethnicities and insurance status. The primary outcome was timely initiation of any first‐course therapy (either radical prostatectomy, radiation therapy, or ADT) within 180 days from date of diagnosis.^23^, ^24^

### 2.2 Statistical methods

Descriptive statistics were used to present baseline characteristics as the NCDB registry captures data from the first course of treatment. Covariates included age (<60, 60–69, and ≥70 years old), clinical T stage (T1, T2, T3, and T4), prostate specific antigen (PSA) level (<10, 10–20, and >20), Gleason score (6, 7, and, 8–10), diagnosis year (2010–2013 vs. 2014–2016), race or ethnicity (White, Black, Hispanic, and Other or Unknown), hospital setting (academic vs. non‐academic), insurance type (not insured, Medicaid, Private, Medicare, or other Government), Charlson–Deyo comorbidity index (0 and ≥1), US region (Northeast, Central, South, and West), household income (<$38,000, $38,000–$47,999, $48,000–$62,999, and ≥$63,000), average education level of zip code where patient is from (≥21.0%, 13–20.9%, 7–12.9%, and <7% of residents with less than high school education), and distance from home to treatment facility (<25, 25–50, and >50 miles). Categorical variables were compared between groups via chi‐squared test and continuous variables via analysis of variance. We used a multivariable logistic regression model to assess the effect of race and insurance type on receipt of definitive therapy within 180 days from date of diagnosis. The final model was built by backward variable selection procedure with an alpha level of 0.10 for removal. The association of race with timely treatment initiation was compared across multiple subgroups, including patients diagnosed before and after 2014, as well as within men with private or Medicare insurance versus men with Medicaid or without insurance. Finally, Cochran–Armitage trend test was performed to assess year‐to‐year changes in proportion of patients with treatment delay >180 days stratified by race. Joinpoint regression was performed at the junction of 2010–2013 and 2014–2016, corresponding to intervals before and after full implementation of the ACA, to assess for significant changes in crude rates of patients with major delay amongst all patients, followed by White and Black patients, respectively. Statistical analysis was performed using SAS 9.4 (SAS Institute Inc.). Two‐sided *p* < 0.05 was considered statistically significant.

## 3 RESULTS

### 3.1 Association of clinicodemographic variables with time to treatment initiation

There were 422,506 men with intermediate or high risk localized prostate cancer diagnosed between 2010 and 2016 pursuing definitive treatment with primary surgery or radiotherapy. 311,398 patients started first‐course treatment (e.g., surgery, radiotherapy, or ADT) within 90 days of diagnosis (73.7%), 92,388 patients started treatment between 91 and 180 days of diagnosis (21.9%), and 18,720 patients started treatment >180 days after diagnosis (4.4%). By race or ethnicity, 6.9% of Black men, 6.1% of Hispanic men, 5.4% of men with other or unidentified race/ethnicity, and 3.8% of White men started treatment >180 days after diagnosis. Distribution of clinicodemographic variables and time to treatment initiation are summarized in Table 1.

**TABLE 1:** Clinicodemographic variables and their association with time from prostate cancer to treatment initiation for patients with localized disease.

		Time from diagnosis to treatment initiation				
Covariate	Level	Median (IQR)	<90 days *n* = 311,398	91–180 days *n* = 92,388	>180 days n = 18,720	Parametric *p*‐value
Age	<60	67 (45–98)	78,546 (25.2%)	27,337 (29.6%)	5617 (30.0%)	<0.001
	60–69	65 (42–96)	13,7206 (44.1%)	44,758 (48.5%)	9054 (48.4%)	
	≥70	50 (22–82)	95,646 (30.7%)	20,293 (22.0%)	4049 (21.6%)	
Prostate specific antigen level	<10	66 (44–96)	184,514 (59.3%)	61,306 (66.4%)	11,595 (61.9%)	<0.001
	10–20	63 (40–96)	50,568 (16.2%)	16,145 (17.5%)	3808 (20.3%)	
	>20	45 (11–78)	76,316 (24.5%)	14,937 (16.2%)	3317 (17.7%)	
T‐Stage	T1	63 (40–95)	187,452 (63.7%)	59,209 (66.8%)	12,327 (69.0%)	<0.001
	T2	62 (39–91)	93,545 (31.8%)	26,861 (30.3%)	5058 (28.3%)	
	T3	50 (28–80)	11,757 (4.0%)	2453 (2.8%)	445 (2.5%)	
	T4	10 (0–39)	1411 (0.5%)	99 (0.1%)	24 (0.1%)	
Gleason score	6	65 (34–102)	45,986 (15.8%)	16,263 (18.2%)	4508 (24.9%)	<0.001
	7	68 (45–98)	166,923 (57.2%)	59,336 (66.5%)	11,588 (64.0%)	
	8–10	50 (28–76)	78,773 (27.0%)	13,656 (15.3%)	2006 (11.1%)	
Race or ethnicity	Others/Unknown	63 (38–97)	11,693 (3.8%)	3734 (4.0%)	887 (4.7%)	<0.001
	Hispanic	65 (37–101)	13,072 (4.2%)	4651 (5.0%)	1150 (6.1%)	
	Black	69 (41–105)	44,124 (14.2%)	17,605 (19.1%)	4568 (24.4%)	
	White	61 (37–90)	242,509 (77.9%)	66,398 (71.9%)	12,115 (64.7%)	
Hospital setting	Non‐academic*	57 (33–86)	200,963 (64.5%)	48,436 (52.4%)	9623 (51.4%)	<0.001
	Academic	70 (46–103)	110,435 (35.5%)	43,952 (47.6%)	9097 (48.6%)	
Charleson‐Deyo Comorbidity Score	≥1	60 (34–91)	60,852 (19.5%)	170,70 (18.5%)	3487 (18.6%)	<0.001
	0	62 (38–93)	250,546 (80.5%)	75,318 (81.5%)	15,233 (81.4%)	
Insurance	Not insured	68 (39–108)	4657 (1.5%)	1840 (2.0%)	563 (3.1%)	<0.001
	Medicaid	69 (40–109)	8126 (2.7%)	3220 (3.6%)	987 (5.4%)	
	Private	65 (43–95)	14,0424 (46.0%)	45,156 (50.2%)	8368 (45.9%)	
	Medicare	56 (30–88)	145,808 (47.8%)	36,802 (40.9%)	7476 (41.0%)	
	Other government	74 (44–114)	6313 (2.1%)	2977 (3.3%)	858 (4.7%)	
Geographical region	Northeast	68 (42–99)	59,663 (19.2%)	21,780 (23.6%)	4365 (23.3%)	<0.001
	Central	57 (35–85)	89,000 (28.6%)	20,819 (22.5%)	3817 (20.4%)	
	South	62 (36–92)	11,6253 (37.3%)	34,747 (37.6%)	6983 (37.3%)	
	West	64 (40–97)	46,482 (14.9%)	15,042 (16.3%)	3555 (19.0%)	
Residence type	Urban/metropolitan	62 (38–92)	297,089 (97.8%)	88,446 (98.3%)	17,933 (98.3%)	<0.001
	Rural	56 (31–84)	6727 (2.2%)	1527 (1.7%)	303 (1.7%)	
Income	<$38,000	61 (35–93)	49,873 (16.1%)	14,509 (15.8%)	3395 (18.2%)	<0.001
	$38,000–$47,999	60 (35–91)	68,777 (22.1%)	19,105 (20.7%)	3822 (20.5%)	
	$48,000–$62,999	62 (38–92)	83,386 (26.8%)	24,325 (26.4%)	4776 (25.6%)	
	≥$63,000	64 (41–94)	108,657 (35.0%)	34,206 (37.1%)	6659 (35.7%)	
Percent of zip code with less than high school education	≥21.0%	62 (35–95)	46,484 (15.0%)	14,295 (15.5%)	3377 (18.1%)	<0.001
	13%–20.9%	62 (36–93)	74,825 (24.1%)	22,104 (24.0%)	4750 (25.4%)	
	7%–12.9%	62 (38–92)	102,673 (33.0%)	30,170 (32.7%)	5740 (30.7%)	
	<7%	63 (40–92)	86,886 (28.0%)	25,635 (27.8%)	4804 (25.7%)	
Distance traveled to treatment	<25 miles	61 (35–91)	226,107 (72.7%)	63,179 (68.5%)	13,027 (69.8%)	<0.001
	25–50 miles	63 (39–92)	42,911 (13.8%)	12,812 (13.9%)	2536 (13.6%)	
	>50 miles	69 (46–101)	41,980 (13.5%)	16,259 (17.6%)	3114 (16.7%)	
Year of diagnosis	2010–2013	61 (36–91)	183,638 (59.0%)	51,253 (55.5%)	10,477 (56.0%)	<0.001
	2014–2016	64 (40–96)	127,760 (41.0%)	41,135 (44.5%)	8243 (44.0%)	

* Non‐academic centers grouped as either community or comprehensive community centers.

On multivariable analysis, non‐White compared with White race was associated with higher odds of treatment delay >180 days. Compared with White men, Black men (OR 1.79, 95% CI 1.72–1.87, *p* < 0.001), Hispanic men (OR 1.37, 95% CI 1.28–1.48, *p* < 0.001), and men with other or unidentified race/ethnicity (OR 1.23, 95% CI 1.14–1.33, *p* < 0.001) had significantly greater odds of experiencing a treatment delay >180 days. Compared to uninsured patients, those with private insurance (OR 0.57, 95% CI 0.52–0.63, *p* < 0.001) or Medicare (OR 0.64, 95% CI 0.58–0.70, *p* < 0.001) had significantly lower odds of major treatment delay; however, patients with Medicaid had no significant difference in odds of treatment delay (OR 0.94, 95% CI 0.84–1.06, *p* = 0.307) compared to uninsured patients. Finally, diagnosis after 2014, was associated with significantly higher odds of major treatment delay (OR 1.11, 95% CI 1.07–1.15, *p* < 0.001). Complete univariable and multivariable associations of clinicodemographic factors with major treatment delays are shown in Table 2. On multiple associations testing, the odds of non‐White patients compared to White patients experiencing major delay was significantly lower after 2014 (OR 1.48; 95% CI 1.41–1.56, *p* < 0.001) compared to before 2014 (OR 1.70; 95% CI 1.62–1.78, *p* < 0.001) with Interaction *p*‐value <0.001.

**TABLE 2:** Univariate and multivariable logistic regression analysis comparing associations of clinicodemographic covariates with proportion of patients experiencing major delay in time from diagnosis to treatment initiation (>180 days).

		Univariate analysis			Multivariable analysis		
Covariate	Level	*n*	Odds ratio (95% CI)	*p*‐value	*n*	Odds ratio (95% CI)	*p*‐value
Age	<60	111,500	‐	‐	95,674	‐	‐
	60–69	191,018	0.94 (0.91–0.97)	<0.001	167,241	1.02 (0.98–1.07)	0.24
	≥70	119,988	0.66 (0.63–0.69)	<0.001	106,223	0.83 (0.79–0.88)	<0.001
Prostate specific antigen level	<10	257,415	‐	‐	233,512	‐	‐
	10–20	70,521	1.21 (1.17–1.26)	<0.001	65,253	1.09 (1.04–1.13)	<0.001
	>20	94,570	0.77 (0.74–0.80)	<0.001	70,373	0.74 (0.71–0.78)	<0.001
T‐Stage	T1	258,988	‐	‐	239,221	‐	‐
	T2	125,464	0.84 (0.81–0.87)	<0.001	115,399	0.82 (0.79–0.85)	<0.001
	T3	14,655	0.63 (0.57–0.69)	<0.001	13,308	0.79 (0.71–0.87)	<0.001
	T4	1534	0.32 (0.21–0.48)	<0.001	1210	0.60 (0.39–0.94)	0.025
Gleason score	6	66,757	‐	‐	58,930	‐	‐
	7	237,847	0.71 (0.68–0.73)	<0.001	222,323	0.61 (0.58–0.63)	<0.001
	8–10	94,435	0.30 (0.28–0.32)	<0.001	87,885	0.28 (0.26–0.29)	<0.001
Race or ethnicity	Others/unknown	16,314	1.47 (1.37–1.57)	<0.001	14,141	1.23 (1.14–1.33)	<0.001
	Hispanic	18,873	1.65 (1.55–1.76)	<0.001	15,838	1.37 (1.28–1.48)	<0.001
	Black	66,297	1.89 (1.82–1.95)	<0.001	58,076	1.79 (1.72–1.87)	<0.001
	White	321,022	‐	‐	281,083	‐	‐
Hospital setting	Non‐academic*	259,022	‐	‐	226,345	‐	‐
	Academic	163,484	1.53 (1.48–1.57)	<0.001	142,793	1.41 (1.37–1.46)	<0.001
Charleson‐Deyo comorbidity score	≥1	81,409	0.96 (0.92–0.99)	0.023	72,281	0.99 (0.95–1.03)	0.497
	0	341,097	‐	‐	296,857	‐	‐
Insurance	Not insured	7060	‐	‐	6014	‐	‐
	Medicaid	12,333	1.00 (0.90–1.12)	0.944	11,184	0.94 (0.84–1.06)	0.307
	Private	193,948	0.52 (0.48–0.57)	<0.001	171,739	0.57 (0.52–0.63)	<0.001
	Medicare	190,086	0.47 (0.43–0.52)	<0.001	170,981	0.64 (0.58–0.70)	<0.001
	Other government	10,148	1.07 (0.95–1.19)	0.259	9220	1.19 (1.06–1.35)	0.004
Geographical region	Northeast	85,808	0.93 (0.89–0.97)	0.001	76,393	0.84 (0.80–0.88)	<0.001
	Central	113,636	0.60 (0.57–0.63)	<0.001	100,904	0.59 (0.56–0.62)	<0.001
	South	157,983	0.80 (0.77–0.83)	<0.001	134,256	0.70 (0.67–0.73)	<0.001
	West	65,079	‐	‐	57,585	‐	‐
Residence Type	Urban/metropolitan	403,468	‐	‐	361,288	‐	‐
	Rural	8557	0.79 (0.70–0.89)	<0.001	7850	0.91 (0.80–1.03)	0.118
Income	< $38,000	67,777	1.13 (1.08–1.18)	<0.001	59,892	0.89 (0.83–0.95)	<0.001
	$38,000–$47,999	91,704	0.93 (0.90–0.97)	<0.001	80,784	0.88 (0.83–0.93)	<0.001
	$48,000–$62,999	112,487	0.95 (0.92–0.99)	0.01	98,766	0.92 (0.88–0.96)	<0.001
	≥$63,000	149,522	‐	‐	129,696	‐	‐
Percent of zip code with less than high school education	≥21.0%	64,156	1.30 (1.24–1.36)	<0.001	55,867	1.12 (1.04–1.19)	0.001
	13%–20.9%	101,679	1.15 (1.10–1.20)	<0.001	88,986	1.12 (1.06–1.19)	<0.001
	7%–12.9%	138,583	1.01 (0.97–1.05)	0.549	122,190	1.03 (0.99–1.08)	0.184
	<7%	117,325	‐	‐	102,095	‐	‐
Distance traveled to treatment	<25 miles	302,313	‐	‐	52,422	1.18 (1.13–1.24)	<0.001
	25–50 miles	58,259	1.01 (0.97–1.06)	0.633	51,956	1.04 (0.99–1.09)	0.083
	>50 miles	61,353	1.19 (1.14–1.24)	<0.001	264,760	‐	‐
Year of diagnosis	2010–2013	245,368	‐	‐	211,655	‐	‐
	2014–2016	177,138	1.09 (1.06–1.13)	<0.001	157,483	1.11 (1.07–1.15)	<0.001

* Non‐Academic centers grouped as either community or comprehensive community centers.

### 3.2 Trends in treatment initiation time after diagnosis over time

**FIGURE 1:**
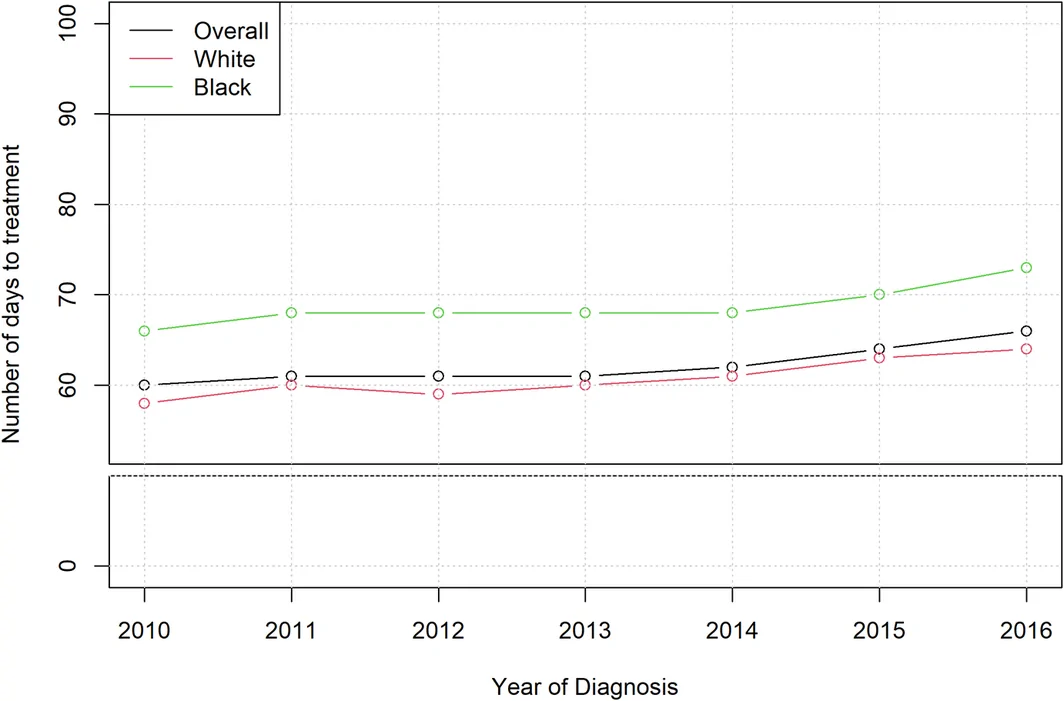
Mean number of days from prostate cancer diagnosis to treatment initiation by year (2010–2016) stratified by race.

**FIGURE 2:**
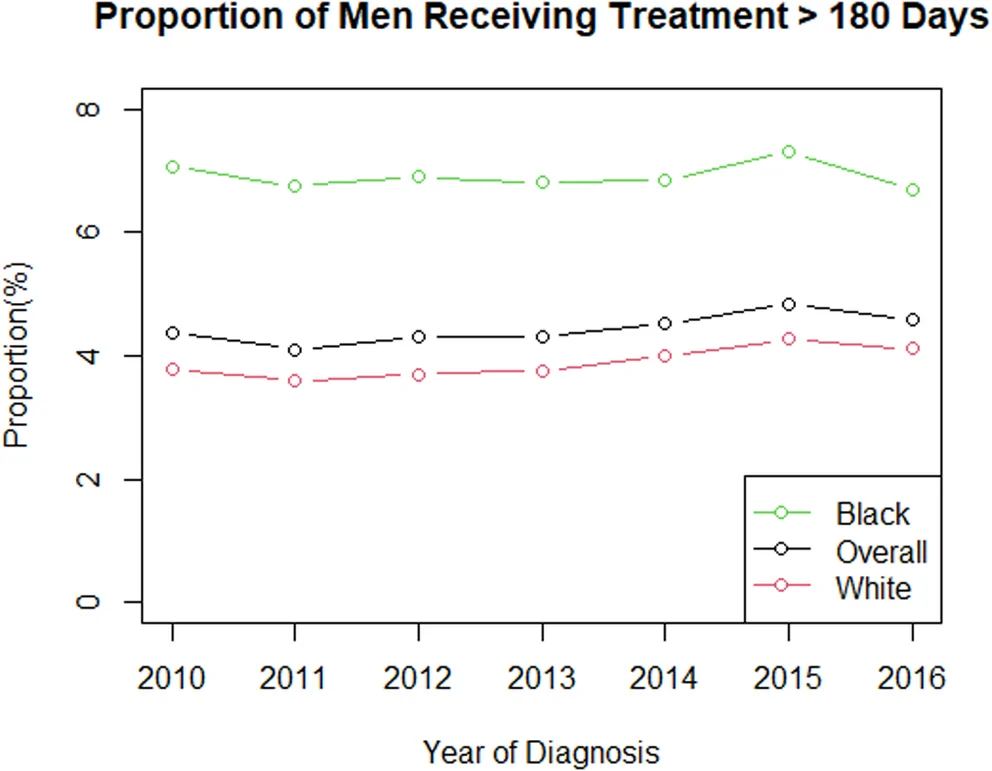
Proportion of patients with at least 180‐day delay from diagnosis to treatment initiation by year (2010–2016) stratified by race.

From 2010 to 2016, mean time from diagnosis to treatment initiation increased for all patients, regardless of race (Figure 1). Mean time from diagnosis to treatment initiation was consistently higher for Black men compared to White men. Median and interquartile range of number of days from diagnosis to treatment for the entire cohort is shown in Figure S2 with median time to treatment increasing from 60 days in 2010 to 66 days in 2016. The proportion of men with major treatment delay >180 days was higher for Black men compared to White men in every year from 2010 to 2016 (Figure 2). From 2010 to 2016, the proportion of Black patients experiencing major treatment delay decreased from 7.1% to 6.7%, while the proportion of White patients experiencing a major treatment delay increased from 3.8% to 4.1%. The number of patients included in this cohort treated per year is shown in Table S1.

Within the entire cohort, the joinpoint between 2010–2013 and 2014–2016 was statistically significant for inflection between crude rates of patients experiencing >180‐day delay from diagnosis to treatment initiation (Figure S3). Additionally the joinpoint between 2010–2013 and 2014–2016 was significant for the subgroup of Black men (Figure S4) as well as for the subgroup of White men (Figure S5). Year‐over‐year, there was a significant trend for increasing time to treatment initiation for the entire cohort (trend *p* < 0.001). This trend remained significant among White men (*p* < 0.001), but not Black men (*p* = 0.98).

### 3.3 Interaction of insurance provider and ACA on associations with delays in treatment initiation

To further assess the impact of health insurance on timeliness of treatment during the study period before and after ACA was operationalized, we examined factors associated with major delay only in patients who had Medicare or private insurance. Within this subgroup, non‐White patients had significantly higher odds of major treatment delay on multivariable analysis, including those who were Black (OR 1.88, 95% CI 1.80–1.96, *p* < 0.001), Hispanic (OR 1.44, 95% CI 1.34–1.55, *p* < 0.001), or other/unidentified race/ethnicity (OR 1.27, 95% CI 1.17–1.37, *p* < 0.001). Furthermore, diagnosis after full implementation of the ACA in 2014 continued to be associated with higher odds of major treatment delay compared to diagnosis prior to 2014 (OR 1.12, 95% CI 1.09–1.16, *p* < 0.001). Complete univariable and multivariable associations of clinicodemographic factors with major treatment delays in subgroup with Medicare or private insurance shown in Table S2.

To further assess the impact of the implementation of the ACA, we analyzed factors associated with major delay for the subgroup of patients diagnosed in 2014–2016. On multivariable analysis, race and ethnicity continued to be significantly associated with odds of treatment delay. Compared to White patients, Black patients (OR 1.80, 95% CI 1.72–1.88, *p* < 0.001), Hispanic patients (OR 1.38, 95% CI 1.28–1.48, *p* < 0.001), and patients with other/unidentified race/ethnicity (OR 1.24, 95% CI 1.14–1.33, *p* < 0.001) had significantly greater odds of experiencing a treatment delay >180 days. There was no significant difference in odds of major delay between patients who were uninsured versus those with Medicaid coverage (OR 0.96, 95% CI 0.85–1.08, *p* = 0.473). Complete univariable and multivariable model of clinicodemographic factors associated with treatment delays in subgroup of men diagnosed after January 1, 2014 shown in Table S3.

## 4 DISCUSSION

In this large analysis of over 400,000 men with intermediate or high risk localized prostate cancer in the United States, we found that non‐White men have significantly greater odds of a >180‐day delay in time from diagnosis to definitive treatment initiation compared to White men. These results remained consistent even after accounting for insurance status or only assessing the years after the ACA was implemented. Furthermore, patients with Medicaid coverage experienced no significant difference in major treatment delays compared to patients without health insurance, while those with private insurance or Medicare coverage have significantly lower odds of major treatment delay. These disparities remained, but slightly decreased in the years after implementation of the ACA. Despite implementation of drastic health care reformative measures aimed at increasing access and reducing disparities, significant differences in disparate access to prostate cancer care remained for men eligible for curative management.

Interestingly, we found that delays in care increased significantly even after implementation of the ACA, perhaps most substantially for White men. It remains unclear why treatment initiation time may have increased over this period. It is plausible that the relatively rapid increase of 14 million newly insured patients through the ACA preceded expansion of provider or hospital capacity. Initial estimates projected 1.3 million additional Papanicolaou tests for cervical cancer screening and a need for more than 50,000 new primary care providers.^25^, ^26^ This mismatch, particularly in the first few years of the ACA, could at least in part explain why more men after 2014 experienced delay in accessing timely prostate cancer treatment in this analysis. Additionally, increase in time to treatment initiation could reflect need for patients to weigh the nuances of an increasingly varied selection of treatment options.

Nonetheless, the effect of the ACA on equitable healthcare access is unclear. While Medicaid expansion increased access to insurance coverage, the evidence for equitable access to care has been less promising.^27^ For example, expansion of Medicaid in New York, prior to the ACA, did not impact racial disparities in utilization of surgical cancer services.^28^ Furthermore, states with early expansion of Medicaid after the ACA saw a decrease in proportion of minority patients with private insurance.^29^ Based on findings in this analysis, those patients who switch from private insurance to Medicaid may become at risk of significant delay in prostate cancer treatment initiation. Of note, many states had not expanded Medicaid access until after our study period, and 12 states have yet to expand Medicaid access as of 2022. Nonetheless, we found in this analysis no difference in timely access to care for patients with Medicaid even after the ACA became operationalized in 2014.

The public health impact of treatment initiation delays in prostate cancer is questionable. While modest differences in time to treatment initiation may not be impactful for favorable risk prostate cancer due to its insidious natural history, these delays can be significant for men with high‐risk or advanced prostate cancer at exceptionally high risk of metastatic dissemination.^30^ Unfortunately, as the epidemiology of prostate cancer evolves in the United States, the impact of treatment delay could grow. Specifically, since the United States Preventive Services Task Force (USPSTF) recommended against routine PSA screening in 2012, the incidence of advanced prostate cancer has increased by 4% annually and incidence of de novo metastatic disease has increased by 6% annually.^31^ Since then, the USPSTF revised recommendations in 2018 regarding PSA screening, specifically highlighting the importance of shared decision‐making with primary care providers, yet no significant difference in rates of screening have resulted.^32^ If men continue to be diagnosed with more advanced prostate cancer, the risks of delays in treatment initiation may magnify disparate clinical outcomes in the United States.^33^, ^34^

Different healthcare systems have had divergent results in fulfilling pledges to reform and increase equity. Studies from the Veterans Health Administration (VHA) have shown no racial disparities in time from diagnosis to radical prostatectomy or mortality with definitive radiotherapy.^13^, ^35^ However, another study suggests potential over‐treatment of Black men with lower risk disease in parallel to undertreatment of higher risk disease at the VHA.^36^ Concerningly, our results and others show that men managed at Commission on Cancer (CoC)‐accredited centers face significant variation in equitable care. Despite thorough use of quality metrics and data monitoring tools at participating facilities, 39% of facilities had higher rates of curative treatment for White men compared to Black men, but just 1% of facilities had the opposite.^37^ Further, based on an analysis using Surveillance, Epidemiology, and End Results data, disparities in definitive treatment rates could be more profound amongst Black men with low compared to high income.^38^ Beyond the standard of care, non‐White men are significantly underrepresented on clinical trials and have lower utilization of advanced radiotherapy techniques such as proton therapy.^39^, ^40^, ^41^, ^42^, ^43^, ^44^, ^45^ The structural racism that Black patients face across healthcare settings perpetuates the disparate access and outcomes.^12^, ^46^, ^47^ We show that timely access to definitive prostate cancer treatment remains a disproportionate albeit narrowing challenge for non‐White men even in the years after the ACA was implemented.

In addition to structural barriers, there are multiple individual‐level factors affecting men seeking prostate cancer care. Provider mistrust can limit timely access to definitive therapy. One study showed that Black men with newly diagnosed prostate cancer and men with fewer years of formal education had significantly higher levels of medical mistrust.^8^ Additionally, when patient and provider race or ethnicity are concordant, patients report better experiences and are more likely to both visit and adhere to provider recommendations.^48^, ^49^, ^50^ Unfortunately, Black and Hispanic providers are underrepresented relative to the general population with minimal increases in the last two decades.^51^, ^52^ Systematic efforts to increase trust in providers and the healthcare system will help men make complex, highly personalized decisions about management of their prostate cancer and may reduce population‐wide disparities in timely care. Apart from the ACA, structural changes in the health system such as hospital consolidation may have affected where patients seek care, which could in turn affect timely access to screening, diagnosis, and treatment. Additionally, increasing complexity of decision making with the development and use of MRI and genomic risk classifiers could prolong time from initial diagnosis to treatment for all men, while different treatment options could have different inherent delays (e.g., booking operating room time or planning radiotherapy treatment).

This study has multiple limitations to note. First, the NCDB is a hospital‐based cancer registry that captures only patients who are diagnosed or treated at CoC‐accredited facilities and may not generalize to the entire United States. However, given that the NCDB captures 70% of newly diagnosed cancer cases in the United States, we believe that this analysis is a representative reflection of general practice patterns. Second, given the retrospective design using a population‐based database, unmeasured confounders could be imbalanced and affect analyses. By adjusting our analyses for all relevant and available clinical and sociodemographic variables in the NCDB, we attempted to mitigate confounding. However, we cannot rule out that unmeasured patient‐level confounders, including performance status and level of social support, could explain treatment patterns. We were not able to account for performance status beyond what the Charlson–Deyo comorbidity score and social supports are not documented in the NCDB. Third, provider‐specific data, including age, gender, and race, are not available in the NCDB but would be impactful to further assess the effect of provider factors on racial and insurance disparities seen in this analysis. Additionally, changing insurance status or dual‐eligible Medicare‐Medicaid patients are not coded in the NCDB. As only a snapshot is provided, this study does not capture history of Medicaid enrollment, prior uninsured status, or if patients changed to a different insurance status after diagnosis. Fourth, granular prostate cancer characteristics aside from clinical T‐stage, documented PSA, and Gleason score, such as PSA kinetics or detailed histopathologic assessment (e.g., percent positive cores, perineural invasion, etc.), are not available in the NCDB. As such, we are unable to confirm if patients with substantial treatment delay may have been considering active surveillance. We attempted to control for this by excluding men with low‐risk disease and those documented as pursuing active surveillance. Fifth, while there is rigorous auditing of the database, it is possible that dates could be inaccurate.^20^, ^21^ Finally, with more routine use of advanced imaging and genetic testing, there could be increased delays in treatment initiation due to prolonged workup itself as well as increased complexity of decision making as patients are re‐categorized to different risk strata. These nuances are not captured in the NCDB.

## 5 CONCLUSION

In conclusion, this large registry analysis of men with localized, curable prostate cancer in the United States revealed striking racial and insurance disparities in timely access to treatment. Specifically, the impact of race and insurance status were independently associated with longer delays to treatment. Further, these disparities were unaffected by implementation of the ACA in 2014. In fact, in the years after implementation of ACA there were increased delays in treatment initiation for all men, regardless of race. Based on this data, the ACA and accompanying Medicaid expansion may not be modifying timely access to prostate cancer care, which is in line with studies in other cancer types. To increase equitable management of prostate cancer, additional work is needed, particularly exploring the differential effects for patients before and after Medicaid expansion in different states. Data on timely access to care in the years since 2016—particularly with more widespread implementation of Medicaid expansion—will be important to guide policy addressing new or continued barriers for patients. As the epidemiology of newly diagnosed prostate cancer in the United States continues to shift due to tempered screening and the COVID pandemic, further work will be needed to increase equity in prostate cancer care.

## AUTHOR CONTRIBUTIONS

**James R Janopaul‐Naylor:** Conceptualization (equal); data curation (equal); formal analysis (equal); investigation (equal); methodology (equal); project administration (equal); visualization (equal); writing – original draft (lead); writing – review and editing (equal). **Taylor J. Corriher:** Writing – original draft (supporting); writing – review and editing (supporting). **Jeffrey Switchenko:** Data curation (equal); formal analysis (equal); investigation (equal); methodology (equal); resources (equal); software (equal); validation (equal); visualization (equal); writing – review and editing (equal). **Sheela Hanasoge:** Writing – review and editing (supporting). **Ashanda Esdaille:** Investigation (supporting); methodology (supporting); writing – review and editing (supporting). **Brandon A Mahal:** Writing – review and editing (supporting). **Christopher P Filson:** Conceptualization (supporting); investigation (supporting); project administration (supporting); supervision (supporting); writing – original draft (supporting); writing – review and editing (supporting). **Sagar A. Patel:** Conceptualization (lead); data curation (equal); formal analysis (equal); investigation (equal); methodology (equal); project administration (equal); resources (equal); supervision (equal); validation (equal); writing – original draft (equal); writing – review and editing (equal).

## FUNDING INFORMATION

Research reported in this publication was supported in part by the Biostatistics Shared Resource of Winship Cancer Institute of Emory University and NIH/NCI under award number P30CA138292. The content is solely the responsibility of the authors and does not necessarily represent the official views of the National Institutes of Health.

## CONFLICT OF INTEREST STATEMENT

None.

## ETHICS STATEMENT

The study was conducted in accordance with local institutional review board policies, using de‐identified data from the NCDB.

## PATIENT CONSENT STATEMENT

The requirement for patient consent was waived.

## Supporting information


Figure S1.

Figure S2.

Figure S3.

Figure S4.

Figure S5.

Table S1.
Table S2.
Table S3.

## Data Availability

Data are available through an application process to investigators associated with Commission on Cancer‐accredited cancer programs.
