# Supplementary figures and images for: Disparities in time to prostate cancer treatment initiation before and after the Affordable Care Act
Source: Cancer Med. 2023 Aug 3;12(17):18258–68. doi: 10.1002/cam4.6419 (PMC10523962; doi:10.1002/cam4.6419)

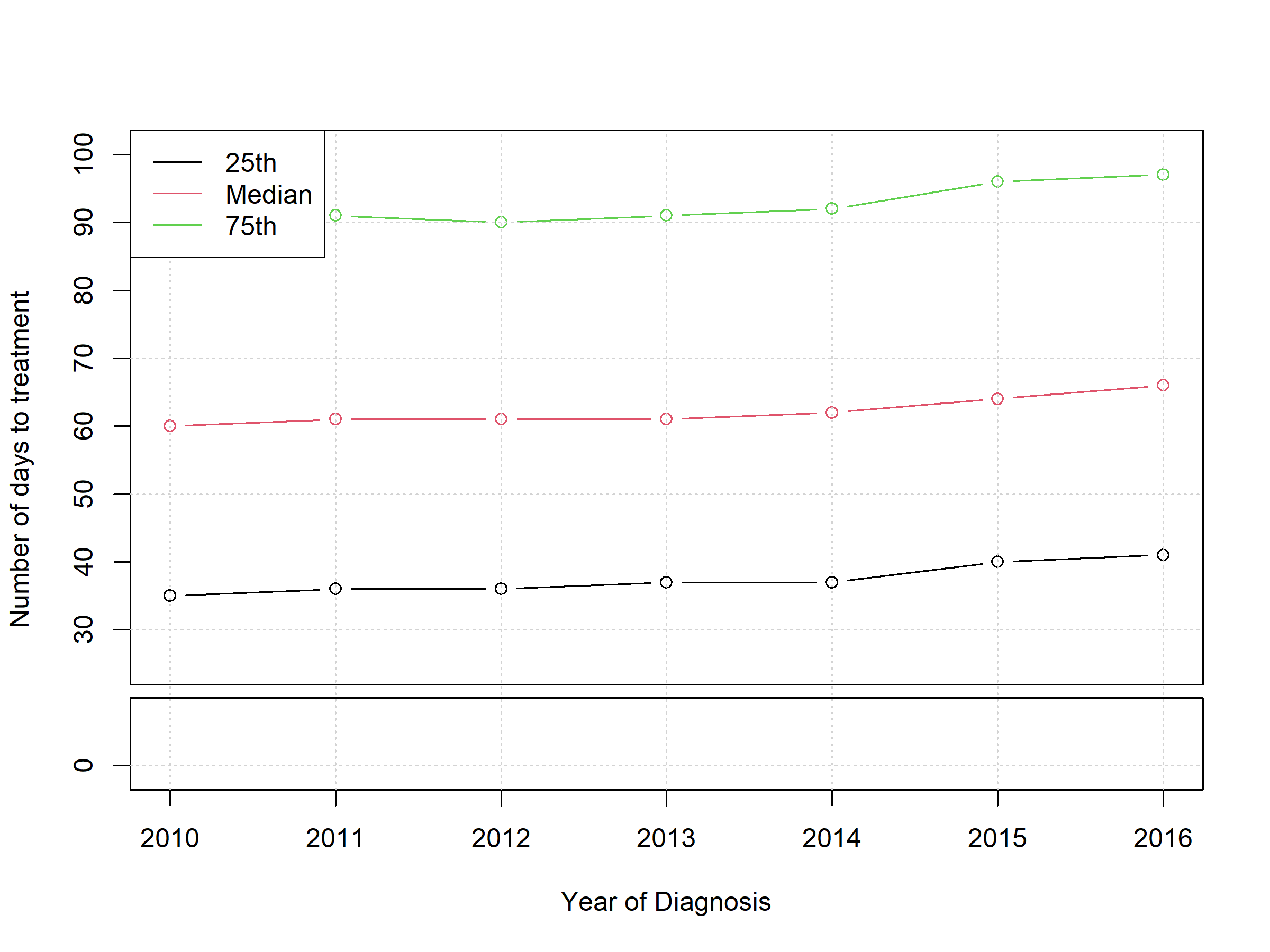

Supplement: Supplementary file 2 — Figure S2. [file CAM4-12-18258-s006.tif]

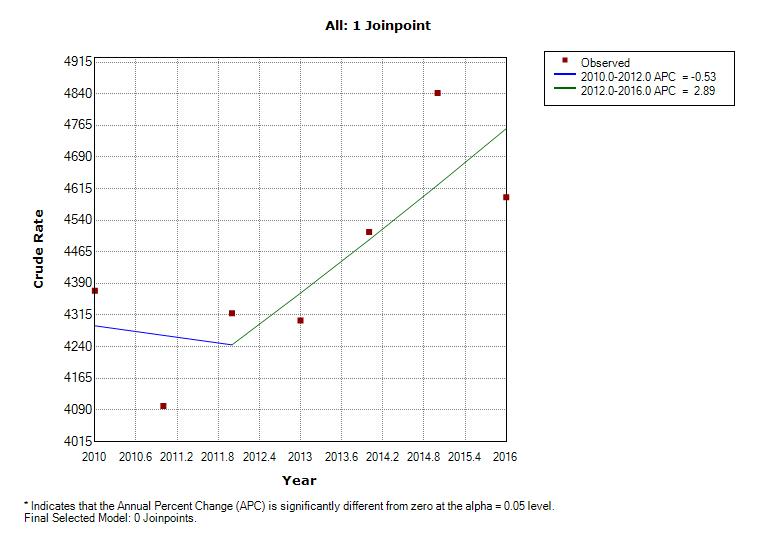

Supplement: Supplementary file 3 — Figure S3. [file CAM4-12-18258-s003.tiff]

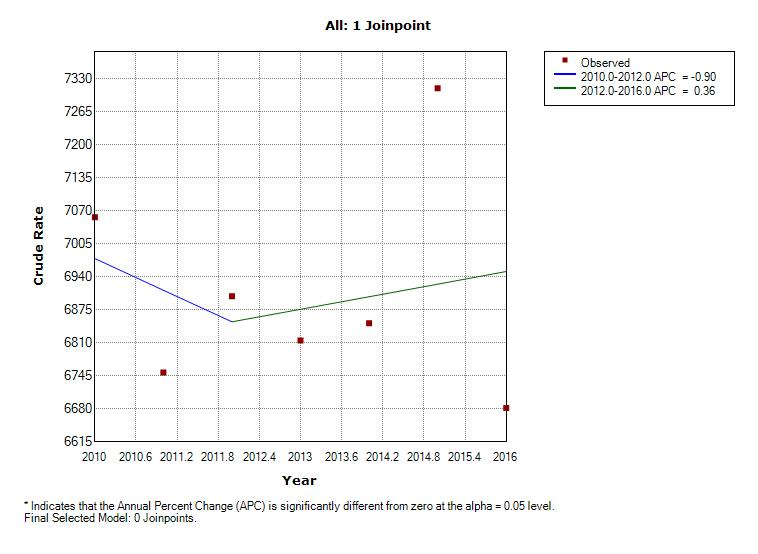

Supplement: Supplementary file 4 — Figure S4. [file CAM4-12-18258-s001.tiff]

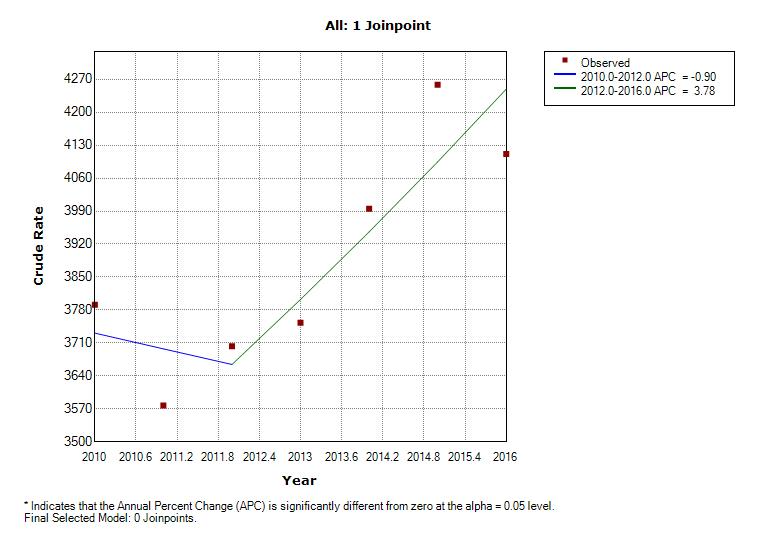

Supplement: Supplementary file 5 — Figure S5. [file CAM4-12-18258-s002.tiff]
